# The Insights of Genomic Synteny and Codon Usage Preference on Genera Demarcation of *Iridoviridae* Family

**DOI:** 10.3389/fmicb.2021.657887

**Published:** 2021-03-31

**Authors:** Zhaobin Deng, Jun Wang, Wenjie Zhang, Yi Geng, Mingde Zhao, Congwei Gu, Lu Fu, Manli He, Qihai Xiao, Wudian Xiao, Lvqin He, Qian Yang, Jianhong Han, Xuefeng Yan, Zehui Yu

**Affiliations:** ^1^Laboratory Animal Center, Southwest Medical University, Luzhou, China; ^2^Department of Anatomy and Embryology, Faculty of Medicine, University of Tsukuba, Tsukuba, Japan; ^3^School of Comprehensive Human Sciences, Doctoral Program in Biomedical Sciences, University of Tsukuba, Tsukuba, Japan; ^4^Key Laboratory of Sichuan Province for Fishes Conservation and Utilization in the Upper Reaches of the Yangtze River, Neijiang Normal University, Neijiang, China; ^5^School of Basic Medical Sciences, Zunyi Medical University, Zunyi, China; ^6^College of Veterinary Medicine, Sichuan Agricultural University, Chengdu, China

**Keywords:** *Iridoviridae*, synteny analysis, taxonomy, codon usage, phylogenetic analysis

## Abstract

The members of the family *Iridoviridae* are large, double-stranded DNA viruses that infect various hosts, including both vertebrates and invertebrates. Although great progress has been made in genomic and phylogenetic analyses, the adequacy of the existing criteria for classification within the *Iridoviridae* family remains unknown. In this study, we redetermined 23 *Iridoviridae* core genes by re-annotation, core-pan analysis and local BLASTN search. The phylogenetic tree based on the 23 re-annotated core genes (Maximum Likelihood, ML-Tree) and amino acid sequences (composition vector, CV-Tree) were found to be consistent with previous reports. Furthermore, the information provided by synteny analysis and codon usage preference (relative synonymous codon usage, correspondence analysis, ENC-plot and Neutrality plot) also supports the phylogenetic relationship. Collectively, our results will be conducive to understanding the genera demarcation within the *Iridoviridae* family based on genomic synteny and component (codon usage preference) and contribute to the existing taxonomy methods for the *Iridoviridae* family.

## Introduction

Members within the family *Iridoviridae* (recently designated iridovirids to avoid confusion between members of this family and members of the genus *Iridovirus* with the same name) are nucleocytoplasmic, large double-stranded DNA (dsDNA) viruses, with diameter ranging from 120 to 300 nm and encoding between 100 and 200 putative proteins; morphologically, they are characterized by a DNA-protein core surrounded by an internal lipid membrane and an icosahedral protein capsid (Chinchar et al., 2017b). Owing to their wide distribution in nature, a large and diverse array of vertebrates and invertebrates can be infected by iridovirids, including insects, amphibians, reptiles and fish (King et al., 2011). The clinical manifestations of iridovirid infections vary greatly from mild symptoms to death, depending on the species. The high morbidity and mortality exhibited by certain species, such as the mandarin fish (*Siniperca chuatsi*), have severely impacted modern aquaculture (Liu et al., 2018). Human activities can also facilitate the spread of certain infectious diseases, as seen in the case of the amphibian population, where tiger salamanders and Chinese giant salamanders (*Andrias davidianus*) have been affected (Whittington et al., 2010; Geng et al., 2011).

With the identification of SHIV and CQIV (Xu et al., 2016; Qiu et al., 2017), as well as the phylogenetic analysis of the new isolates, a new genus (*Decapodiridovirus*) was established, leading to an increase in the number of genera under the family *Iridoviridae* from five to six. This includes *Iridovirus*, *Chloriridovirus* and *Decapodiridovirus* which infect invertebrates, *Lymphocystivirus* and *Megalocytivirus* which only affect bony fish, and *Ranavirus* which has a wide host spectrum (fish, amphibians and reptiles) (Chinchar et al., 2017a). Initially, the iridovirids were classified based on their particle size, host preference, GC content, the presence of a DNA methyltransferase, as well as the existence of major capsid protein (MCP) (King et al., 2011). With the development of complete genome sequencing, phylogenetic analysis based on pan-genomic data provided an insight into the criteria for virus taxonomy. As a result, a set of essential genes conserved among all viruses in the family *Iridoviridae* was defined (Eaton et al., 2010). However, while only limited sequences of iridovirids have been obtained, new iridovirids are still continuously being discovered, thus making it difficult to distinguish and classify the viruses precisely. Additionally, even though the core set of genes was conserved, some unique genes only exist within specific species (Eaton et al., 2007). Furthermore, it is still not clear whether the current criteria are sufficient to determine the genera of newly discovered viruses. Thus, deeper and more comprehensive insights into the existing classification approach are urgently needed for a more comprehensive and objective differentiation.

Owing to the degeneracy of the genetic code, an amino acid may correspond to more than one codon; therefore, most of the codons are synonymous, while the frequency of occurrence of those are unbalanced among varied genes and most of organisms, which is referred as codon usage bias (Ikemura, 1981, 1985; Chen et al., 2014). Codon usage preference is a widespread phenomenon in nature and can be found in viruses, prokaryotes, eukaryotes and even in different genes within the same organism (Greenbaum et al., 2008; Rahman et al., 2018). An increasing number of studies have suggested that codon usage is influenced by multiple factors, such as mutation pressure, natural selection, GC content, tRNA abundance and protein secondary motifs, among others. Of these factors, the former two play a crucial role (Cristina et al., 2015; Wang et al., 2016; Ur Rahman et al., 2017; Rahman et al., 2018). The codon usage pattern has a strong connection with virus survival, adaptation, evolution and the control of host immune system (Butt et al., 2014; Rahman et al., 2018). Studying this codon usage bias may thus provide more information regarding virus molecular evolution, providing a further insight into virus taxonomy and phylogenetic analysis.

In the present study, phylogenetic analysis, synteny analysis and comprehensive codon usage analysis were performed based on a total of 53 iridovirids. We found that the genomic synteny relationship and codon preference within the family *Iridoviridae* may provide a new reference factor for the virus classification.

## Materials and Methods

### Sequence Data Retrieval

The complete genome sequences of 53 iridovirids spanning 39 years from 1979 to 2018 used in this study (Supplementary Table 1) were retrieved from the National Center for Biotechnology (NCBI) GenBank database^1^. Detailed information of these viruses is listed in Supplementary Table 1, including the sequence name, host information, country of origin, year of isolation and accession number.

### Virus Annotation and Detection of Core Genes

The annotation of obtained virus genome was performed using Prokka [version 1.14.5; Seemann (2014); settings: –kingdom Viruses, remaining settings: default]. Core genes, which are highly conserved and shared by all iridovirids (Eaton et al., 2007; Jancovich et al., 2010), play a fundamental role in revealing the phylogenetic relationship among the species. With the emergence of high-throughput sequencing, there has been a paradigm shift in microbial genomics studies to a large-scale pan-genome analysis. Thus, the pan-genome detection of core genes was preferentially performed by PanX (Ding et al., 2018) for the published 53 completed iridovirids genomes (settings: –ngbk, –cg 0.9, –nsl, which mean sequence identity threshold and disable long branch splitting, respectively; remaining settings: default). Meanwhile, the remaining core genes were manually screened by local BLASTN search against the GenBank database.

### Phylogenetic Analysis

Composition vector phylogenetic tree (CV-Tree) is considered a faithful and an objective method to deduce evolutionary relatedness and has previously been successfully applied to viruses, chloroplasts and fungi (Wu et al., 2017; Mao and Wang, 2019). Furthermore, it also has the advantages of being whole-genome-based and alignment free. To gain insight into the genetic variability and evolution of the viruses in different genera, the CVTree web server was employed according to the user’s manual. Briefly, FASTA formatted files containing all the genomic amino acid sequences were directly submitted to CVTree web server (version 3^2^) and the *K*-value was set at 5. Subsequently, Evolview v3 (Subramanian et al., 2019) was recruited for the visualization and annotation of the phylogenetic tree.

Prior to the construction of the phylogenetic tree, different multiple sequence alignments based on the core genes of the iridovirids were generated by MUSCLE program (version 3.8.31) (Edgar, 2004). Subsequently, the aligned sequences were submitted to PhyloSuite (Zhang et al., 2020) for concatenation. Finally, Maximum Likelihood (ML) phylogenetic trees were constructed using MEGA 6.0.

### Synteny Analysis

Synteny analysis was implemented using MCScanX (Wang et al., 2012) for all iridovirid genera, including *Iridovirus*, *Chloriridovirus*, *Decapodiridovirus*, *Lymphocystivirus*, *Megalocytivirus*, and *Ranavirus*. Prior to analysis, the amino acid sequences of each virus was compared against itself and other members using BLASTP (version 2.8.1). The acquired results were then filtered according to identity >50%. Finally, the dot-plotter picture was drawn (refer to the software manual)^3^.

### Codon Preference Indices

#### Relative Synonymous Codon Usage

Relative synonymous codon usage (RSCU) was first proposed by Sharp and Li (1986) and helps to remove the influence caused by amino acid composition on codon usage. Thus, it has been widely used for evaluating the codon usage preference between genes. To investigate the codon usage bias pattern of indicated viruses, the codon nucleotide sequences of core genes were subjected to software CodonW. Meanwhile, the RSCU values of all codons in them were calculated. A RSCU value higher or lower than 1.0 would suggest a positive or negative bias toward that codon, respectively. In contrast, a RSCU value that is nearly equal to 1.0 indicates that the codons were chosen equally and randomly.

#### Correspondence Analysis

Correspondence analysis (COA), a type of multivariate statistical analysis, not only displays the sets of rows and columns in specific dataset with geometrical representation (Wong et al., 2010), it also shows the major variable trends and helps to detect the relationships between variables and samples. In this study, COA based on RSCU values was performed by means of the CodonW software. Briefly, each coding region of the virus was represented as a 59-dimensional vector and every dimension corresponds to the RSCU value for each codon (excluding AUG, UGG and stop codons). The visualized graphics were drawn by R ggplot2 package.

#### ENC-Plot Analysis

Effective number of codons (ENC) was proposed by Wright (1990) and has been widely used to determine the codon bias. The ENC value ranges from 20 (only one specific codon is recruited for each amino acid) to 61 (the recruitment percentage for all synonymous codons is equal). ENC-plot (ENC vs CG3s) is an efficient and visual method to determine whether the codon usage bias is caused by mutation only (the corresponding points would lie on or be close to the standard curve) or by multiple factors such as natural selection (points would be distributed away from the expected curve). The expected ENC values were calculated following the method documented by Singh et al. (2016).

#### Neutral Evolution Analysis

Neutral evolution analysis (Neutrality plot analysis) was used to determine and compare between the extent of the influence exerted by natural selection and mutation pressure on the codon usage patterns of coding segments within the genera. Briefly, the GC_12_ values of synonymous codons were plotted against the GC_3_ values (Butt et al., 2016). The values of GC_12_ and GC_3_ of iridovirids were calculated by the CodonW program and subjected to neutrality plot analysis.

#### Parity Rule 2 Plot Analysis

The Parity rule 2 (PR2) plot analysis was used to identify the effects of natural selection and mutation on codon usage bias, which is characterized by the value of AT-bias [A_3_/(A_3_ + T_3_)] as the ordinate and GC-bias [G_3_/(G_3_ + C_3_)] as the abscissa (Sueoka, 1995). A_3,_ T_3,_ G_3_ and C_3_ correspond to the A, T, G, and C content at the third position of four-codon amino acids, respectively. In this plot, the center, where both coordinates are 0.5 (means A = T and G = C), indicates that mutation and selection have an equal effect on codon usage (Sueoka, 1995, 1999).

## Results

### Detection of Conserved Genes in Iridovirids

To clarify the genetic composition of 53 members from the family *Iridovirdae* and normalize the differences caused by various sequencing and annotation platforms, Prokka program was employed. After re-annotation, core-pan analysis and BLASTN search were conducted for the detection of core genes within the *Iridovirdae* family. As a result, a total of 26 *Iridoviridae* core genes were detected in most of the 53 iridovirids [the *Iridoviridae* core genes were identified by Eaton et al. (2007)], although only 23 of them were shared by all 53 iridovirids. The genes coding for ribonucleotide reductase small subunit and proliferating cell nuclear antigen were absent within CQIV and SHIV (CQIV_MF197913/SHIV_MF599468) and serine-threonine protein kinase was absent within AMIV (AMIV_KF938901).

### Phylogenetic Analysis

**FIGURE 1:**
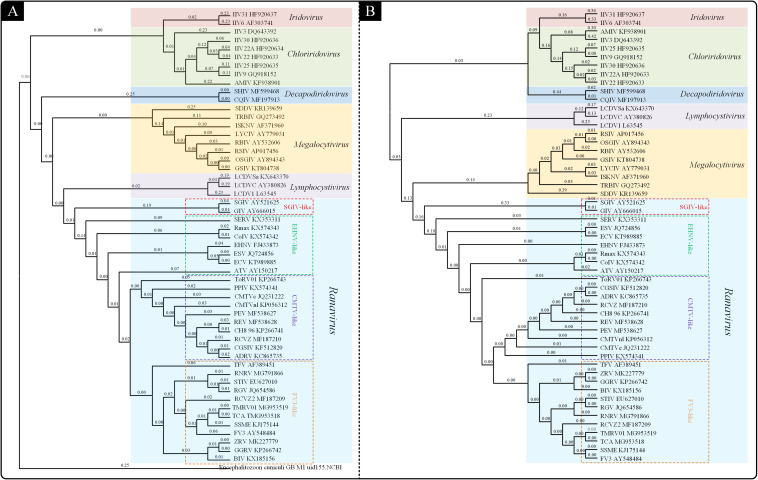
Phylogenetic analysis of the *Iridoviridae* family. **(A)** Phylogenetic tree based on genomic amino acid sequences constructed using composition vector method (CV-Tree). **(B)** Phylogenetic tree based on the 23 *Iridoviridae* core genes constructed using maximum likelihood method (ML-Tree).

To understand the probable genetic relationships within the *Iridoviridae* family, a genome-wide phylogenetic analysis was performed by using CVTree web server based on genomic amino acid sequences of the *Iridoviridae* family members (Figure 1A). The result (CV-Tree) showed that the 53 viruses could be classified into the 6 genera: *Iridovirus*, *Chloriridovirus*, *Decapodiridovirus*, *Megalocytivirus*, *Lymphocystivirus*, and *Ranavirus*. The genera *Ranavirus* could be further divided into four subgroups: SGIV-like, EHNV-like, CMTV-like and FV3-like.

Owing to the different alignment methods or insufficient data, the branching order of the *Iridoviridae* family is often inconsistent between genomic papers (Jancovich et al., 2003; Huang et al., 2009). Thus, to clarify the evolutionary relationships within the family *Iridoviridae*, a phylogenetic analysis (ML-Tree) based on the core-genes was conducted (Figure 1B). The resulting ML-phylogenetic tree exhibited similar distribution pattern of the given viruses, as well as a similar subgroup classification within Ranaviruses.

### Synteny Analysis

**FIGURE 2:**
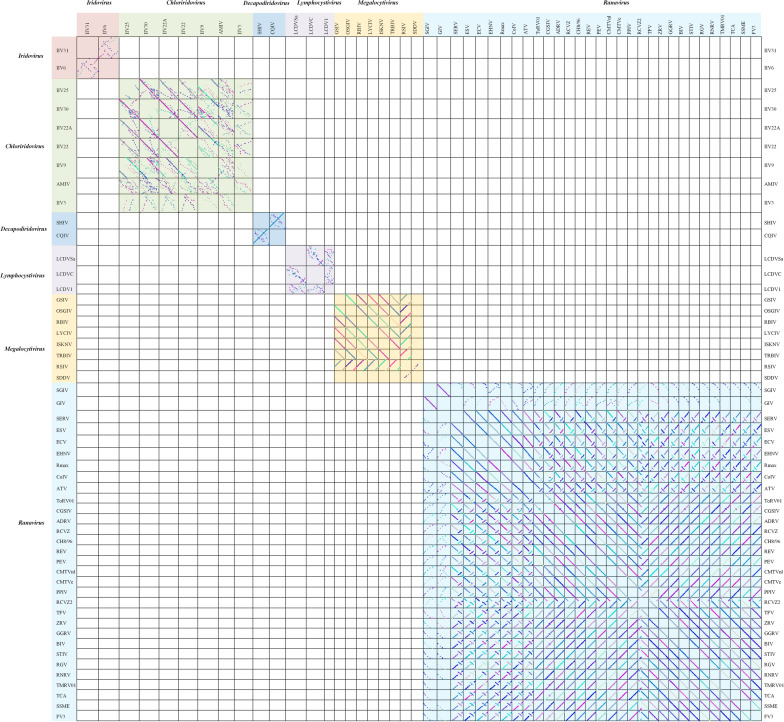
The dot-plotter analysis of 53 members of the *Iridoviridae* family (identity > 50%).

To clarify the linear relationships of the *Iridoviridae* family members, MCScanX was employed to obtain a full landscape of the 53 virus genomes. After screening (identity threshold set as 50%), the dot-plotter results showed that members within the same genus shared certain regions of collinearity as compared to those in different genera and there was an obvious demarcation between the different genera (Figure 2). The specific collinearity in each genus was summarized in Table 1.

**TABLE 1:** The specific collinearity in the indicated genera.

	Shared longer regions	Shared fragmentary regions	Reason
*Iridovirus*	–	IIV31 and IIV6	IIV6 and IIV31 are distantly related
*Chloriridovirus*	Except for AMIV and IIV3	AMIV and IIV3	Both AMIV and IIV3 have a distant relationship as compared to other members
*Decapodiridovirus*	SHIV and CQIV	–	Close relationship between SHIV and CQIV
*Lymphocystivirus*	–	LDVSa, LCDVC, and LCDV1	LDVSa, LCDVC and LCDV1 are distantly related
*Megalocytivirus*	Except for SDDV	SDDV	Distant relationship between SDDV and other members
*Ranavirus*	Except for GIV and SGIV	GIV and SGIV	Both GIV and SGIV have a distant relationship as compared to other members

### Codon Usage Preference Analysis

#### Relative Synonymous Codon Usage Analysis

**FIGURE 3:**
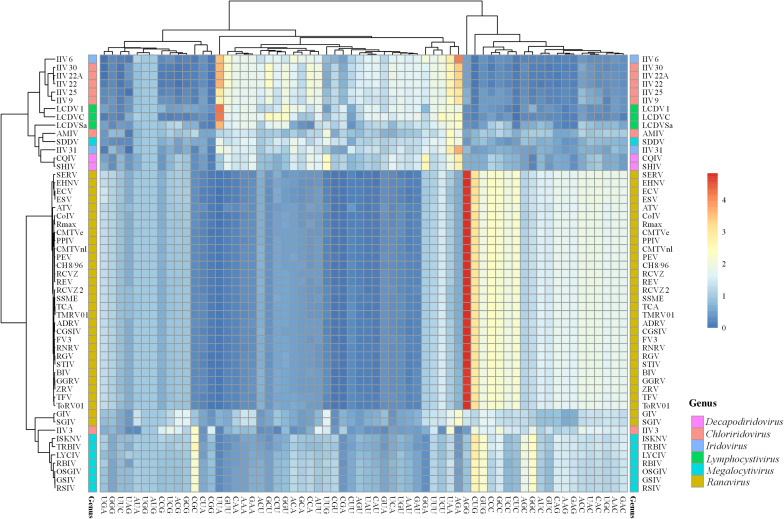
Heat map of RSCU values of 53 iridovirids. Each column represents a codon and each row represents a different virus species.

To clarify the patterns of synonymous codon usage of members in *Iridoviridae* family, relative synonymous codon usage (RSCU) analysis of 59 codons was performed to describe the codon usage bias among different genera. All values and large variances obtained were highlighted in the Supplementary Table 1 and were further corroborated by the summary of the hierarchical clustering of RSCU values (Figure 3). Among all the codons, UUU (Phe) was the most common one that was shared by all 53 iridovirids. Viruses in the genus *Decapodiridovirus* were found to prefer U- and A- ending codons and the RSCU values of CQIV and SHIV were identical. Similarly, viruses in *Iridovirus*, also exhibited a preference for U- and A- ending codons. Although, the topology of the cluster analysis of these viruses was generally consistent with the results of the phylogenetic analysis, there were still some interesting observations. For example, AMIV and IIV3 that were clustered into the same group in the ML-phylogenetic tree exhibited two different codon usage bias patterns, of which a total of 11 amino acids (Leu, Val, Ser, Pro, Thr, Tyr, Gln, Lys, Glu, Arg, and Gly) were involved. In addition, the codon usage bias of GIV and SGIV was slightly different as compared to other members in the *Ranavirus* genus. For example, GIV and SGIV preferred using AGA (RSCU values: 2.22 and 2.24) to encode for arginine, while the other members preferred AGG. Similar differences were also observed in the codon choices for Leu, lle, Ser, Thr, Ala, Gln, Lys, Glu, and Gly. Different preferences in codon usage within the same genus may suggest that the virus codon preference is not fully consistent with phylogenetic analysis.

#### Correspondence Analysis

**FIGURE 4:**
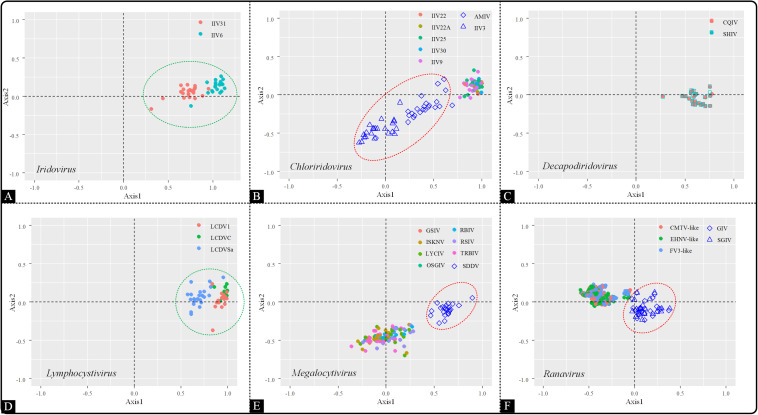
Correspondence analysis of the synonymous codon usage in the *Iridoviridae* family. The analysis was based on the RSCU value of the 59 synonymous codons. Green circles suggest a high dispersion degree among the points inside the circle, while red circles indicate that points inside the circle have a high degree of dispersion as compared to those outside the circle. **(A)** *Iridovirus*, **(B)** *Chloriridovirus*, **(C)** *Decapodiridovirus*, **(D)** *Lymphocystivirus*, **(E)** *Megalocytivirus*, and **(F)** *Ranavirus.*

As a multivariate statistical method, correspondence analysis (COA) can minimize the effect caused by amino acid composition and reduce the dimensions of datasets to obtain awareness of multiple variables. To detect the trends in codon usage variation, COA was implemented based on RSCU values. The genera *Iridovirus*, *Decapodiridovirus* and *Lymphocystivirus* displayed similar distribution pattern, with all the members in each genus forming a main cluster (Figures 4A,C,D). However, in the *Chloriridovirus* genus, AMIV and IIV3 were widely spread in the second, third and fourth quadrants, unlike other members which were grouped together (Figure 4B). Interestingly, a similar phenomenon was observed for *Megalocytivirus* and *Ranavirus*. Specifically, SDDV in *Megalocytivirus* formed an isolated cluster away from other *Megalocytivirus* members (Figure 4E). Similarly, the distribution of the synonymous codon usage patterns in *Ranavirus* showed a division into two clusters, one composed of GIV and SGIV and the other formed by the remaining viruses, including CMTV-like, EHNV-like, and FV3-like (Figure 4F). These results were consistent with the landscape presented in the ML-phylogenetic tree (Figure 1B), indicating that codon usage bias may reflect evolutionary relationships to some extent.

#### ENC-Plot Analysis

**FIGURE 5:**
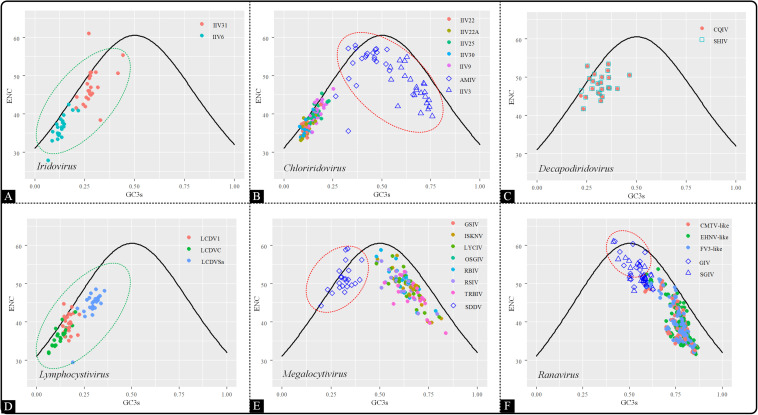
ENC–GC3 plot of 53 *Iridoviridae* viruses. The solid line indicates the expected curve of ENC vs GC3 only under mutational pressure. Points on or close to the expected curve mean that the bias is caused by mutation pressure, while points below the curve indicate the presence of other influential factors such as natural selection. Green circles suggest a high dispersion degree among points inside the circle, while red circles indicate that points inside the circle have a high degree of dispersion as compared to those outside the circle. **(A)** *Iridovirus*, **(B)** *Chloriridovirus*, **(C)** *Decapodiridovirus*, **(D)** *Lymphocystivirus*, **(E)** *Megalocytivirus*, and **(F)** *Ranavirus.*

To investigate the factors affecting codon usage patterns among the coding sequences in the *Iridoviridae* family, an ENC-GC3 plot was generated. This was used to evaluate the codon usage pattern is dominated by mutational bias or selection pressure (Wright, 1990; Wu et al., 2015). In the ENC-GC3 plot, the points that lie on or close to the curve represent that the genetic evolution is only affected by mutational pressure. Conversely, the points below the curve indicate that the codon usage pattern is still subjected to natural selection. The results showed that the majority of the points fell beneath the expected curve, except a few points that were located on or close to the curve, indicating that overall, codon usage bias was also shaped by the presence of natural selection rather than by mutational pressure alone (Figure 5). Interestingly, the distribution pattern of *Chloriridovirus*, *Megalocytivirus* and *Ranavirus* were consistent with the COA results and their points were spread out while being clearly divided into two clusters (AMIV and IIV3 vs others; SDDV vs others; GIV and SGIV vs others). Thus, it is reasonable to infer that codon usage pattern is associated with evolutionary relationships.

#### Neutrality Plot Analysis

**FIGURE 6:**
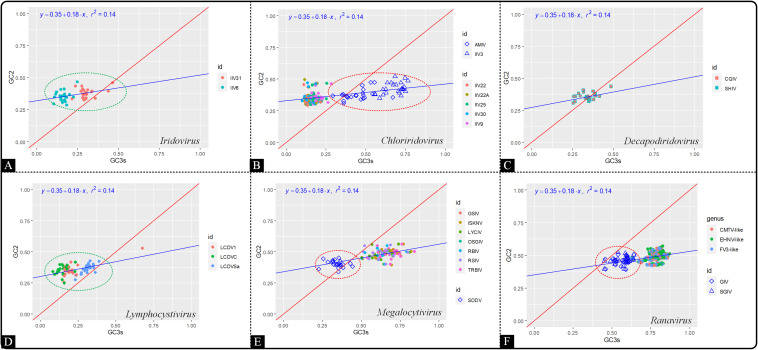
Neutrality plot analysis based on GC12 against GC3 of 53 *Iridoviridae* viruses. The blue line is the linear regression of GC12 against GC3 (*r*^2^ = 0.14). Green circles suggest a high dispersion degree among points inside the circle, while red circles indicate that points inside the circle have a high degree of dispersion as compared to those outside the circle. **(A)** *Iridovirus*, **(B)** *Chloriridovirus*, **(C)** *Decapodiridovirus*, **(D)** *Lymphocystivirus*, **(E)** *Megalocytivirus*, and **(F)** *Ranavirus.*

In order to investigate the contribution of mutation pressure and natural selection, a neutrality plot analysis was produced based on GC12 against GC3. The neutrality plot revealed that the correlation between GC12 and GC3 values among all the genera of the *Iridoviridae* family was relatively low (*r*^2^ = 0.14) (Figure 6). The slope of regression line was calculated to be 0.18, indicating that mutation pressure and natural selection accounted for 18 and 82%, respectively. Thus, both mutation bias and natural selection had influence over the codon usage pattern and the latter was dominant. In addition, viruses in the genera *Choriridovirus*, *Megalocytivirus* and *Ranavirus* clearly gathered into two clusters, which was consistent with the results of the COA and ENC-GC3 plot.

#### PR2 Plot

**FIGURE 7:**
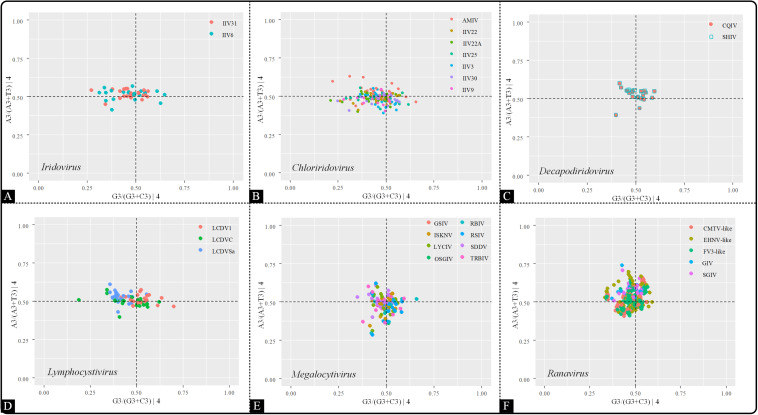
PR2 plot analysis of the viruses within the indicated genera. The positions of the points were based on the AT and GC bias in the third codon position. **(A)** *Iridovirus*, **(B)** *Chloriridovirus*, **(C)** *Decapodiridovirus*, **(D)** *Lymphocystivirus*, **(E)** *Megalocytivirus*, and **(F)** *Ranavirus.*

To further analyze the influence of highly biased gene restriction on codon usage pattern, PR2 bias plots were conducted by comparing the relationships between the AT and GC contents in four-codon sequences (Ala, Arg, Gly, Leu, Pro, Ser, Thr, and Val). The center of the plot (both coordinates are 0.5) is the place where A = T and C = G, suggesting that the impact of mutation pressure and natural selection are equal. In the present study, A_3_/(A_3_ + T_3_) and G_3_/(G_3_ + C_3_) were plotted as the ordinate and abscissa, respectively (Figure 7). Across all six genera, the points were clustered into one group and also spread out in all four quadrants (A≠T, G≠C), denoting the presence of different driving forces other than mutation pressure, such as natural selection, and that the influence of each factor was not equal.

## Discussion

Initially, owing to the characteristics of highly conserved variable domains, the major capsid protein (MCP) was thought to be reliable for the evolutionary analysis of iridovirids (Tidona et al., 1998). However, with the deepening of our understanding of iridovirid genomics, there has also been increasing evidence that phylogenetic analysis based solely on MCP may not be sufficiently comprehensive. Developments in the evolutionary analysis of iridovirids were made in 2007 following the re-analysis of 12 different genomes that found 26 core-set genes across all iridoviruses (Eaton et al., 2007). These core genes are typically conserved and associated with the steps involved in virus infection such as virulence, replication and gene expression (Eaton et al., 2007; Jancovich et al., 2015). Owing to the common access to the genome sequences of iridovirids, information from newer studies about molecular function could be updated over time, and may provide additional insights into phylogenetic analysis. Some core genes might have been exchanged during evolution, bringing the number of core genes down to 24. For example, genes coding for deoxynucleotide reductase and small subunit of ribonucleotide reductase were absent in European Chub *Iridovirus* and Shrimp hemocyte iridescent virus, respectively (Qiu et al., 2018). Interestingly, in the present study, only 23 core genes were shared by all the iridovirids studied. Furthermore, the phylogenetic analysis based on these core genes showed a similar map of genus demarcation as compared with previous reports (Chinchar et al., 2017b; İnce et al., 2018). Thus, it is reasonable to infer that the definition of core genes might need to be re-considered.

Currently, the co-linear arrangement analysis among viruses is mainly dependent on Java-Dotter (JDotter), a widely used method to visualize linear relationships and genomic structural changes. However, it is best utilized with a limited number of virus samples and would be time-consuming for a sample size of more than 10. Consequently, in order to obtain an all-inclusive understanding of collinear relationship among 53 iridovirids, BLASTP and MCScanX were employed. According to the description of ICTV (file code: 2018.007D), the members between different genera should share less than 50% amino acid sequence identity. MCScanX can visualize this genus demarcation criteria by the preliminary screening (identity > 50%). Briefly, the corresponding box would be blank (both viruses share less than 50% identity) for those viruses failed screening (Figure 2). Collectively, MCScanX shows more advantages as compared to Java-Dotter, such as by being faster, capable of analyzing more samples (over 50) and allowing for visualization of the genus demarcation criteria. To the best of our knowledge, this is the first study that applied MCScanX in the analysis of virus genomes.

According to the description of ICTV (file code: 2018.007D), the species demarcation mainly depends on following features: genomic component (genomic size and G+C content), phylogenetic relatedness and a co-linear arrangement of genes. However, the information provided by these features is relatively limited. In current study, we found that the characteristics of codon usage preference can provide more evidence, which were consistent with the results of phylogenetic and synteny analysis. As a result, the 53 iridovirids from 6 genera can be divided into the following categories: first, *Decapodiridovirus*, where members shared similar content and low degree of dispersion (COIV and SHIV); second, *Iridovirus* and *Lymphocystivirus*, where members shared different content and high degree of dispersion (IIV31 and IIV6; LDVSa, LCDVC, and LCDV1); third, *Chloriridovirus*, *Megalocytivirus* and *Ranavirus*, where members shared different content and high degree of dispersion as compared to their counterparts within the same genus (AMIV and IIV3; SDDV; GIV and SGIV). Interestingly, codon usage preference was consistent with the results of phylogenetic and synteny analysis (Table 2), which provides more information about genomic component than genomic size or G+C content. Thus, it is likely that the discovery of this phenomenon will be able contribute to the existing research on virus taxonomy.

**TABLE 2:** Comparison of codon usage preference, synteny analysis and phylogenetic analysis between different genera in the family *Iridoviridae.*

	Codon usage preference, (Neutrality plot COA, ENC-plot)	Synteny analysis	Phylogenetic analysis
*Decapodiridovirus*	Low dispersion between COIV and SHIV	COIV and SHIV shared longer regions	COIV and SHIV showed short branch length
*Iridovirus*	High dispersion between IIV31 and IIV6	IIV31 and IIV6 shared fragmentary regions	IIV31 and IIV6 showed long branch length
*Lymphocystivirus*	High dispersion among LDVSa, LCDVC and LCDV1	LDVSa, LCDVC and LCDV1 shared fragmentary regions	–
*Chloriridovirus*	High dispersion between AMIV and IIV3 and other counterparts	AMIV and IIV3 shared fragmentary regions compared with other members	AMIV and IIV3 showed long branch length
*Megalocytivirus*	High dispersion between SDDV and other counterparts	SDDV shared fragmentary regions compared with other members	SDDV showed long branch length
*Ranavirus*	High dispersion between GIV and SGIV and other counterparts	GIV and SGIV shared fragmentary regions compared with other counterparts	GIV and SGIV showed long branch length

## Data Availability Statement

The datasets presented in this study can be found in online repositories. The names of the repository/repositories and accession number(s) can be found in the article/Supplementary Material.

## Author Contributions

ZY, ZD, JW, and WZ: conceptualization. ZY, ZD, JW, WZ, and YG: methodology. ZY, YG, and MZ: software. CG, LF, MH, QX, and WX: formal analysis. LH, QY, JH, and XY: investigation. ZY and ZD: writing–original draft preparation. JW and WZ: writing–review and editing. ZY: funding acquisition. All authors contributed to the article and approved the submitted version.

## Conflict of Interest

The authors declare that the research was conducted in the absence of any commercial or financial relationships that could be construed as a potential conflict of interest.

## Footnotes

1 http://www.ncbi.nlm.nih.gov

2 http://tlife.fudan.edu.cn/cvtree/cvtree/

3 https://github.com/wyp1125/MCScanX
